# Fetal Brain Development in Congenital Heart Disease

**DOI:** 10.1016/j.cjca.2022.09.020

**Published:** 2022-09-27

**Authors:** Shabnam Peyvandi, Caitlin Rollins

**Affiliations:** a University of California San Francisco Benioff Children’s Hospital, San Francisco, California, USA; b Boston Children’s Hospital and Harvard University Departments of Neurology, Boston, Massachusetts, USA

## Abstract

Neurodevelopmental impairments are the most common extracardiac morbidities among patients with complex congenital heart disease (CHD) across the lifespan. Robust clinical research in this area has revealed several cardiac, medical, and social factors that can contribute to neurodevelopmental outcome in the context of CHD. Studies using brain magnetic resonance imaging (MRI) have been instrumental in identifying quantitative and qualitative difference in brain structure and maturation in this patient population. Full-term newborns with complex CHD are known to have abnormal microstructural and metabolic brain development with patterns similar to those seen in premature infants at approximately 34 to 36 weeks’ gestation. With the advent of fetal brain MRI, these brain abnormalities are now documented as they begin *in utero*, as early as the third trimester. Importantly, disturbed brain development *in utero* is now known to be independently associated with neurodevelopmental outcome in early childhood, making the prenatal period an important timeframe for potential interventions. Advances in fetal brain MRI provide a robust imaging tool to use in future neuroprotective clinical trials. The causes of abnormal fetal brain development are multifactorial and include cardiovascular physiology, genetic abnormalities, placental impairment, and other environmental and social factors. This review provides an overview of current knowledge of brain development in the context of CHD, common prenatal imaging tools to evaluate the developing fetal brain in CHD, and known risk factors contributing to brain immaturity.

## RÉSUMÉ

Les troubles neurodéveloppementaux sont parmi les états morbides extracardiaques les plus fréquents dans la vie des patients atteints d’une cardiopathie congénitale (CC) complexe. Des recherches cliniques rigoureuses ont mis en umière différents facteurs cardiaques, médicaux et sociaux qui contribuent aux troubles neurodéveloppementaux en contexte de CC. Des études faisant appel à l’imagerie par résonance magnétique (IRM) du cerveau ont été déterminantes pour caractériser les différences quantitatives et qualitatives dans les structures cérébrales et la maturation du cerveau dans cette population de patients. Les nourrissons nés à terme atteints de CC complexe présentent des anomalies du développement microstructurel et métabolique du cerveau comparables à celles observes chez les bébés prématurés nés à 34 à 36 semaines de gestation. L’arrivée de l’IRM cérébrale foetale permet maintenant de documenter ces anomalies lors de leur apparition in utero, dès le troisième trimestre de grossesse. Il est important de souligner qu’un retard de développement cérébral in utero est un facteur indépendant associé à des troubles neurodéveloppementaux dans la petite enfance. La période prénatale pourrait être une période importante pour de possibles interventions. Les progrès réalisés dans le domaine de l’IRM cérébrale fœtale en font un puissant outil d’imagerie à utiliser lors d’essais cliniques futurs sur la neuroprotection. Les causes des anomalies du développement cérébral fœtal sont multifactorielles et incluent la physiologie cardiovasculaire, les anomalies génétiques, les dysfonctions placentaires et d’autres facteurs environnementaux et sociaux. Notre article fournit un aperçu de l’état actuel des connaissances sur le développement cérébral en contexte de CC, des outils d’imagerie prénatale fréquemment utilisés pour évaluer le développement cérébral fœtal dans les cas de CC et des facteurs de risque connus qui contribuent à l’immaturité cérébrale.

Congenital heart disease occurs at a frequency of 0.3 to 0.6 per 1000 live births.^1^ With advances in surgical techniques and perioperative care, survival has improved considerably for patients with complex congenital heart defects (CHDs), leading to a growing adult population.^2–4^ Although there has been a decline in severe neurologic insults, many patients experience behavioural, emotional, cognitive, and motor impairments, suggesting widespread brain dysfunction.^5–11^ Importantly, neurodevelopmental (ND) impairments are the most common comorbidity that patients with CHD face. Over the past 2 decades, research has focused on understanding brain development, brain injury, and ND outcomes in the CHD population.

It was long thought that the developmental abnormalities in children with CHD were secondary to surgical and perioperative factors such as the need for cardiopulmonary bypass in infancy. However, in one of the first neonatal brain magnetic resonance imaging (MRI) studies performed, Miller et al. observed that neonates with complex forms of CHD have evidence of dysmature brain development even before going to the operating room.^12^ Similar findings were observed utilizing semiquantitative methods of measuring brain development in neonates with CHD.^13^ Subsequently, Limperopoulos et al. observed these same patterns in the third-trimester fetus, providing direct evidence that apparent delays in brain development in complex CHD begin in late gestation.^14^ The divergence from normal begins in the third trimester of fetal life—a time of rapid brain growth and development that presumably requires a significant increase in oxygen and nutrient delivery, which may be challenging in the face of aberrant cardiovascular physiology. In this review, we provide an overview of current knowledge on the developing fetal brain in the context of CHD with the advent of novel MRI techniques. We review the proposed multifactorial etiologies of delayed brain development with contributions from genetic abnormalities, cardiovascular physiology, and environmental factors.

## Brain Development in CHD

In contrast to human cardiac structure, which is largely determined by gestational week 7,^15^ brain structure continues to evolve over a much longer period of time that extends into the third trimester and postnatally. Brain maturation in the third trimester of pregnancy is marked by dynamic processes including neuronal proliferation, axonal growth, oligodendrocyte maturation, and synaptogenesis, yielding a dramatic increase in cerebral volume and neuronal connectivity.^16,17^ Consequently, blood flow to the developing brain increases in the third trimester and is estimated to be one-fourth the combined ventricular output, demonstrating the unique interplay between the heart, circulation, and brain that is critical for normal brain development.^18^

Overall, late-gestation fetuses and newborns with significant CHD have smaller brains with simplified cortical gyrification, less organised white matter tracts, and immature biochemistry and electrical activity, as summarised in Table 1.^12,14,19,20^ Automated and manual volumetry techniques can model brain tissue characteristics and have identified decreased total and regional brain volumes in fetuses and neonates with complex CHD.^14,21^ Other quantitative techniques, such as diffusion tensor imaging (DTI) and spectroscopy, have been applied to the fetus and neonate with CHD. DTI has demonstrated decreased microstructural brain development in newborns with CHD, with higher levels of apparent diffusion coefficient and lower levels of fractional anisotropy.^12^ Similarly, metabolic brain development as measured by metabolic compounds such as *N*-acetyl aspartate–choline ratio is known to be decreased in the fetus and newborn with complex CHD.^12,14^

Qualitative structural abnormalities of the brain are observed in fetuses and neonates with CHD. In a study by Brossard-Racine et al., fetal brain MRI detected structural abnormalities in 23% of CHD fetuses (total studied = 144) compared with 1.5% in healthy fetuses.^22^ The most common abnormalities included mild unilateral ventriculomegaly and increased extra-axial spaces. These structural brain anomalies were not associated with severity of cardiac lesion and occurred in both cyanotic and acyanotic CHD. Interestingly, 4 fetuses had evidence of white matter signal hyperintensity, which may represent a precursor to the typical pattern of white matter injury or periventricular leukomalacia seen on postnatal MRI scans in CHD.

**Figure 1.**
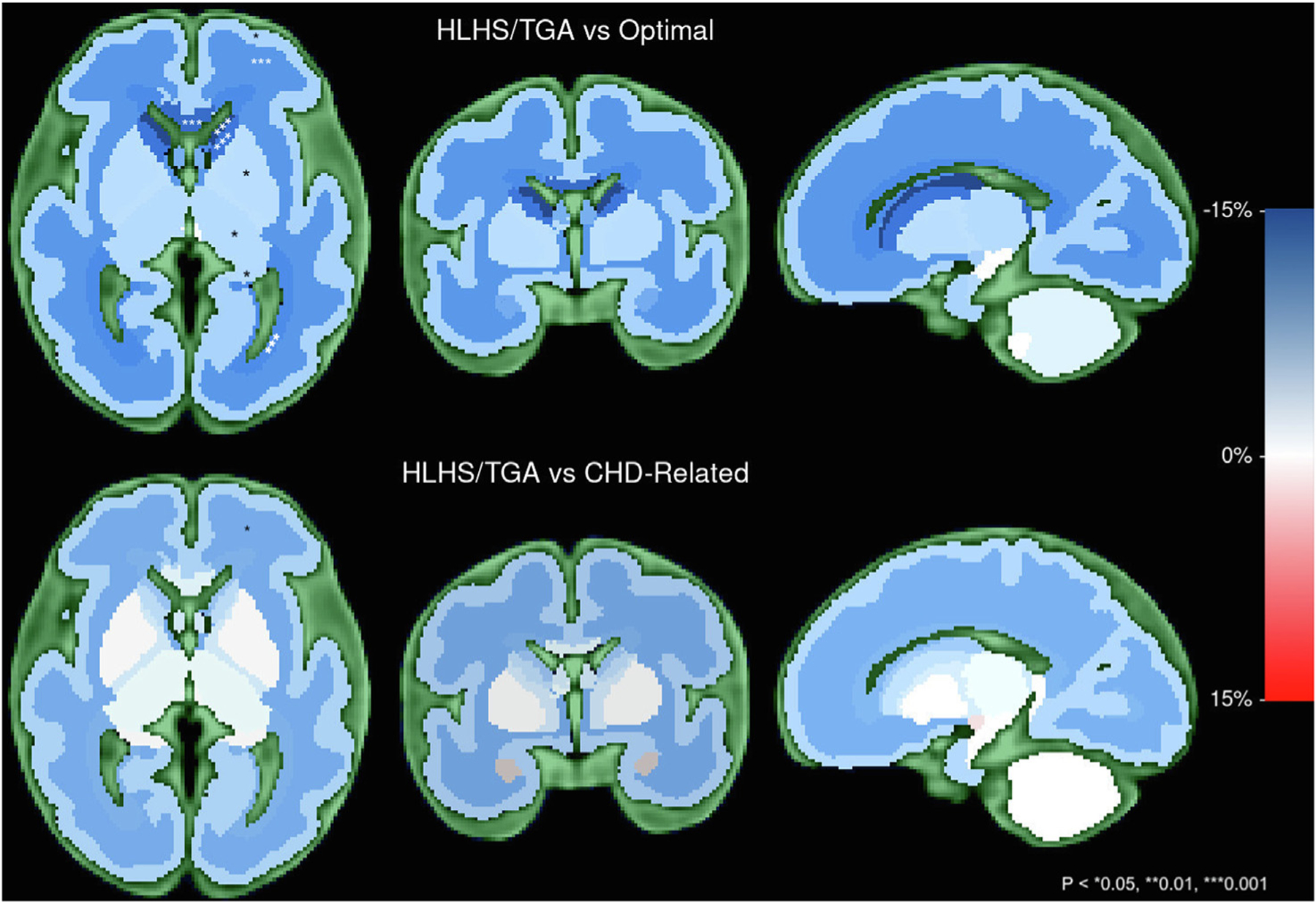
Brain volume differences in fetuses with congenital heart disease compared with healthy fetuses (“optimal”) and CHD-related control fetuses (eg, healthy siblings of patients with known CHD). Estimated group differences in regional brain volumes for fetuses with HLHS or TGA compared with each control group are depicted in (**left**) axial, (**middle**) coronal, and (**right**) sagittal planes in a 32-week gestational age fetal brain. **Blue** indicates a relative reduction in brain volume of the structure compared with the reference group; **red** indicates a relative increase. Intensity reflects the magnitude of the estimated group difference in regional brain volume relative to the reference group for a 32-week gestational age male, according to the scale provided. **Asterisks** denote significance according to false discovery rate—adjusted *P* values comparing group-difference β-estimates (Table 3): *P < 0.05; **P < 0.01; ***P < 0.001. CHD, congenital heart disease; HLHS, hypoplastic left heart syndrome; TGA, dextro-transposition of the great arteries. Modified from Rollins et al.^23^ with permission from John Wiley and Sons.

With advancements in fetal MRI, including technology to account for fetal and maternal motion, important additional details have been observed about brain structure. Rather than a uniform reduction in volume throughout the brain, fetuses with CHD show a region-specific pattern, with regions containing structures vulnerable to low oxygen or substrate delivery most affected. In one fetal brain MRI study, the ventricular, intermediate, and subplate zones, which contain neural progenitor cells, premyelinating oligodendrocytes, and subplate neurons, were smaller in fetuses with CHD than in healthy control subjects (Fig. 1).^23^ These volumetric disturbances were greatest among fetuses with hypoplastic left heart syndrome (HLHS) and dextro-transposition of the great arteries (d-TGA), forms of CHD associated with particularly low *in utero* oxygen and substrate delivery. Similarly, nonhuman animal models of fetal CHD suggest heightened vulnerability in the proliferative regions as well as developing white matter components.^24–26^ Disturbed development in these critical structures important for neuronal proliferation, myelination, and cerebral connectivity may contribute to long-term differences in brain structure and function. Moreover, collectively these findings provide indirect evidence that circulatory abnormalities contribute to disturbed fetal brain development in CHD.

**Figure 2.**
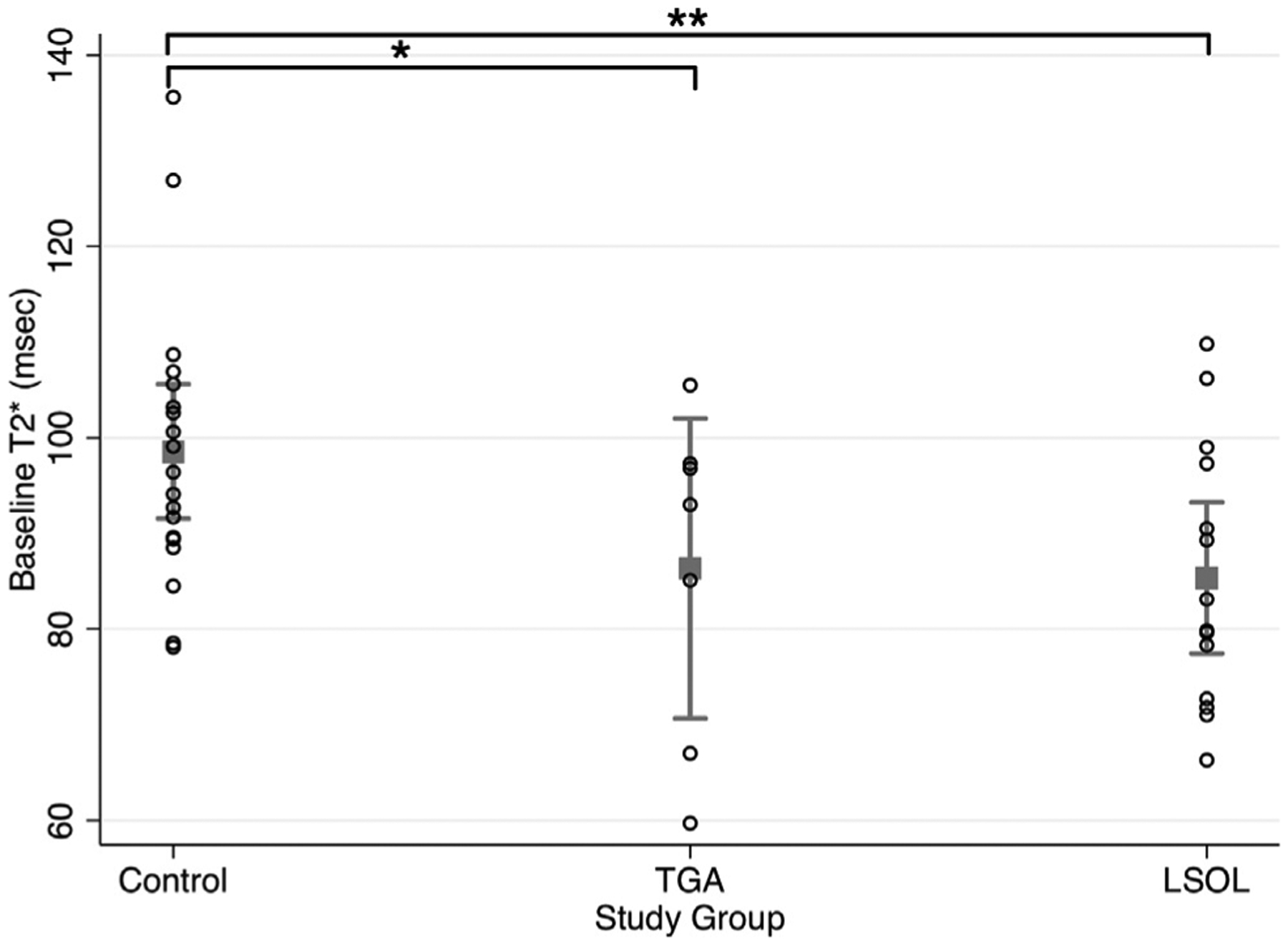
T2* values at baseline for control, LSOL, and TGA groups with mean and 95% confidence intervals (CIs; error bars). At baseline, cerebral tissue oxygenation (T2*) is significantly lower in the LSOL (**coeff: −15.4, 95% CI −25.3 to −5.5; *P* = 0.003) and TGA (*coeff: −12.0, 95% CI −24.4 to 0.4; *P* = 0.05) groups compared with the control group after adjusting for gestational age at MRI. LSOL, left-side obstructive lesion; MRI, magnetic resonance imaging; TGA, dextro-transposition of the great arteries. Modified from Peyvandi et al.^27^ with permission of John Wiley and Sons.

Other quantitative MRI techniques have revealed abnormalities in brain oxygenation. T2* MRI has been widely used in humans for different clinical applications such as functional, susceptibility-weighted, perfusion, and iron-deposition MRI, including studies focused on the brain. Quantitative T2* imaging can estimate fetal blood oxygenation in tissue by measuring relative levels of deoxyhemoglobin. Higher levels of deoxyhemoglobin in the tissue result in faster rates of decay in the T2* signal. T2* reflects a volume-weighted average of both arterial and venous oxygenation in the tissue of interest, thus not measuring specifically oxygen delivery or oxygen consumption but providing an overall assessment of tissue oxygenation. Studies have shown that fetuses with CHD have a faster rate of T2* signal decay than control subjects in the third trimester, indicating lower overall cerebral tissue oxygenation.^27,28^ Interestingly, fetuses with HLHS and d-TGA have similar T2* decay times in the brain (Fig. 2). Although the underlying cardiovascular physiology is significantly different between these 2 groups, these data suggest that both lesions result in overall decreased cerebral oxygenation and metabolism to the same degree. This is in line with other studies demonstrating that these 2 groups of patients have a similar degree of fetal and early neonatal abnormalities in brain development but start to diverge after the neonatal operation.^29^ In particular, those with d-TGA that undergo a corrective operation in the neonatal period, essentially normalising cardiovascular physiology, start to exhibit more rapid brain growth than those with HLHS that undergo a palliative operation with ongoing abnormal cardiovascular physiology. However, recent studies suggest that the perinatal transitional period may result in significant circulatory changes affecting brain health that may differ by underlying cardiovascular physiology. For example, this period is marked by reduced systemic and cerebral perfusion in neonates with HLHS.^30^

Cohort studies beginning in the fetal period have provided insight on trajectories of brain development with the use of imaging as well as the relationship between fetal brain structure and ND outcomes after birth. For example, fetuses with HLHS are now recognised to have a progressive fall-off in cortical and subcortical grey matter, as well as white matter volumes, through the third trimester.^20^ In addition, perinatal impairments in brain growth appear to affect subsequent brain growth trajectories.^19^

Other studies have sought to understand the predictive value of structural brain abnormalities in assessing neonatal structural abnormalities and brain injury in the CHD population.^31^ Brain abnormalities were found in 16% of the fetal brain MRIs and in 32% of the neonatal MRIs. Structural abnormalities seen on the fetal brain MRI included isolated ventriculomegaly, increased extra-axial spaces, white matter cysts, isolated vermian hypoplasia, and white matter signal hyperintensity on T2-weighted images. On the neonatal brain MRI, acquired injury was seen in 26% of the cases, mostly in the form of white matter injury in the periventricular white matter, the centrum semiovale, and the frontal white matter as well as nonhemorrhagic parenchymal injury (ie, focal infarction, diffuse injury, and cysts). Interestingly, of the 33 abnormal neonatal brain MRIs, only 9 were preceded by abnormalities on the fetal brain MRI, resulting in a high specificity (89%) but low sensitivity (27%) of conventional fetal brain MRI in predicting neonatal findings. In addition, 8 subjects had abnormal fetal MRI findings that were not seen on the neonatal MRI. The fetal brain abnormalities that resolved or normalised by the neonatal time period included mild increase in extracerebral space, mild unilateral ventriculomegaly, immature brain appearance, vermian hypoplasia, and single frontal subependymal cyst. That study demonstrated that in addition to prenatal brain immaturity, perinatal and birth-related events contribute and predispose neonates to further cerebral injury after birth.

With advanced fetal MR techniques now able to measure quantitative aspects of brain development, fetal brain volume appears to be an important predictor for postnatal ND outcomes. In a recent study of patients with various forms of CHD, fetal total brain volume predicted 10% to 21% of the variance in ND outcome at age 2 years.^32^ When combining fetal brain volume with data on other known sociodemographic and medical risk factors, models explained up to 45% of variance in ND outcomes. Compared with other sociodemographic and medical data, fetal brain volume was the most consistent predictor of outcome across different domains of development and the only predictor of adaptive functioning. Future larger studies are needed to replicate these findings, but these data provide compelling support for targeted efforts to optimise brain development in the fetal period.

## Genetic factors that contribute to brain development in CHD

Children with CHD and a genetic syndrome are known to have more significant impairments in ND outcomes than children with isolated CHD.^6^ However, recent literature suggests that nonsyndromic genetic differences likely also contribute to overall ND outcome. This topic will be covered in detail in a separate review in this issue of the *Canadian Journal of Cardiology*. Preliminary evidence points toward nonsyndromic genetic differences affecting brain structure and function and thus brain development. In one of the largest multicentre studies performed to date, exome sequencing was performed on more than 1000 children with CHD and their parents (trios).^33,34^ Damaging *de novo* mutations in high heart expression genes were identified in patients with CHD and parent-reported ND disorders. Using these data, a follow-up study identified 12 genes that were affected in this cohort that are also known to contribute to the development of the connectom—dfine connections that develop over time stemming from neurogenesis, dendritic development, and synapse formation.^35^ These data demonstrate that genetic differences contribute to the overall developmental phenotype observed in patients with CHD.

## Cardiovascular factors contributing to brain development in CHD

In the normal fetus, cerebral blood flow is supplied by highly oxygenated blood from the ductus venosus preferentially streaming across the foramen ovale to the left atrium and ventricle. Depending on the subtype of CHD, cerebral blood flow and thus oxygen delivery can be impaired. For example, in d-TGA, the aorta and pulmonary artery are transposed and thus the higher oxygenated blood reaches the pulmonary vasculature instead of the aorta. In hypoplastic left heart syndrome, inadequate left heart structures lead to reversal of blood flow in the foramen ovale with mixing of oxygenated and deoxygenated blood in the right ventricle and, in the cases of aortic atresia, retrograde flow in the ascending aorta. This altered circulation can lead to flow disturbances that may affect *in utero* growth and brain development. In fact, many large studies have revealed that infants with CHD had abnormal *in utero* somatic growth compared with matched control subjects. Specifically, those with d-TGA had normal birth weights for gestational age but small head circumferences relative to birth weight. Those with HLHS were symmetrically small for gestational age with lower birth weights, lengths, and head circumferences.^36^

Multimodal fetal imaging techniques, including ultrasound and MRI, have demonstrated how this altered circulation can lead to flow disturbances affecting *in utero* growth and brain development. Cerebral Doppler ultrasound can assess fetal cerebral vascular resistance in the middle cerebral artery by calculating the pulsatility index or resistance index, a measure of vascular resistance in the circulatory bed downstream from the point of Doppler sampling. Multiple studies have shown that fetuses with HLHS have lower impedance or resistance within the middle cerebral artery, similar to the patterns observed in growth-restricted fetuses in a chronic hypoxic state.^37–39^ Those studies demonstrate that alterations in the intracardiac circulation caused by specific cardiac defects result in changes in cerebral blood flow characteristics. The mechanism is complex and likely related to altered cerebral blood oxygen content as well as possibly decreased blood flow resulting in overall decreased oxygen and nutrient delivery.

The interplay between cardiovascular physiology and brain development has been directly explored using novel fetal cardiac MRI techniques that can quantify flow and oxygen saturations in fetal blood vessels.^40–43^ By combining fetal brain MRI and cardiovascular MRI, Sun et al. found a correlation between fetal cerebral oxygen consumption and brain size (estimated brain weight) among 30 fetuses with CHD in late gestation.^44^ There was a direct correlation between estimated brain weight and cerebral oxygen consumption. In addition, there was a modest association between cerebral oxygen delivery and brain size. These findings suggest that the hemodynamic alterations that occur *in utero* in fetuses with CHD contribute to abnormalities in brain development.

## Environmental factors contributing to brain development in CHD

The developmental origins of health and disease hypothesis (Barker hypothesis) states that a disturbance in environmental factors during critical periods of development has an organisational effect on biological systems, including the central nervous system.^45^ In line with this hypothesis, natural experiment studies and other observational studies have demonstrated a link between prenatal maternal stress and developmental outcomes in offspring without congenital anomalies. For example, a natural experiment study conducted on pregnant mothers during the famous 1998 ice storm in Québec demonstrated that higher levels of prenatal maternal stress were associated with worse cognitive and language outcomes in offspring at 2 years of age in otherwise healthy children.^46^ More recent studies using fetal brain MRI in otherwise healthy fetuses have shown a link between prenatal stress and alterations in the developing connectome, particularly in the amygdala, hippocampus, and cortical volumes.^47,48^ The biological mechanism of these findings is hypothesised to be through epigenetic mechanisms at the level of the placenta acting as a mediator of maternal and environmental signals to the developing fetus.^49,50^ As a mediator of maternal and environmental signals to the developing fetus, epigenetic processes within the placenta can lead to alterations in placental gene expression and signalling that can then lead to changes in developmental programming in the fetus. Nonhuman animal studies have focused on the role of maternal stress and elevated glucocorticoids as one example of environmental stimuli. Typically, the transmission of maternal glucocorticoids across the placenta is prevented by a placental enzyme that converts cortisol to an inactive metabolite. However, in the setting of chronic prenatal maternal stress induced in a rodent model, this enzyme was down-regulated, allowing for unchecked cortisol transmission to the fetus.^51^

Not surprisingly, maternal stress is known to be elevated with a prenatal diagnosis of a complex CHD.^52^ In a cross-sectional study by Yu et al., mothers carrying a fetus with CHD and a group of control subjects in the third trimester (mothers with a normal fetus) were enrolled to compare levels of maternal psychologic distress and whether there were any observed associations with brain development on fetal brain MRI.^53^ Sixty-five percent of mothers carrying a fetus with CHD tested positive for stress, 44% reported anxiety, and 29% reported depression, much higher rates compared with the control subjects. Interestingly, among the CHD group only, maternal stress and anxiety were associated with smaller hippocampal and cerebellum volumes. Thus, maternal stress appears to be associated with fetal brain growth and development in the setting of CHD as well. Studies are currently underway to understand the effects of long-standing stress both *in utero* and after birth on ND outcomes in children with CHD.

As mentioned above, many animal experiments identify the placenta as an important organ in facilitating environmental signals to the developing fetus.^54^ However, placental development and function itself can also be abnormal in the setting of CHD. In fact, conditions thought to result from abnormal placental function, such as preeclampsia, intrauterine growth restriction, and prematurity, are more common in pregnancies affected by CHD.^55^ Placental pathology studies have observed significant abnormalities in the setting of fetal CHD. Common abnormalities on pathologic examination include thrombosis, infarction, immature villi, and abnormal placental perfusion.^56^ The extent to which placental abnormalities precede the development of CHD, placental pathology develops secondary to abnormal cardiovascular physiology, and/or common risk factors (eg, hyperglycemia) contribute to both processes is unclear. Advanced fetal MRI has allowed for increased quantitative measurements of placental size and function.^57^ For example, in a study by You et al., blood oxygen level—dependent MRI was used to assess the response of the placenta to supplemental oxygen administered to the mother.^58^ They found that in fetuses with HLHS, there was an acute increase in placental oxygenation as measured by blood oxygen level—dependent MRI compared with control fetuses. The interpretation of these findings is unclear; however, they do further demonstrate that placental function and physiology are altered in the setting of complex CHD. Related to this concept, studies have demonstrated progressive changes in umbilical artery impedance, with an increase over the course of gestation in pregnancies affected by CHD. This increased placental resistance may reflect altered cardiac output or other circulatory factors that affect the placenta and ultimately the brain. Interestingly, higher umbilical artery pulsatility in the third trimester has been associated with worse ND outcomes in fetuses with CHD.^59^ Finally, shared genetic pathways in placental, cardiac, and brain development involving angiogenesis have been hypothesised to play a role in the pathology observed in these fetuses.^60^

## Future Directions

**Figure 3.**
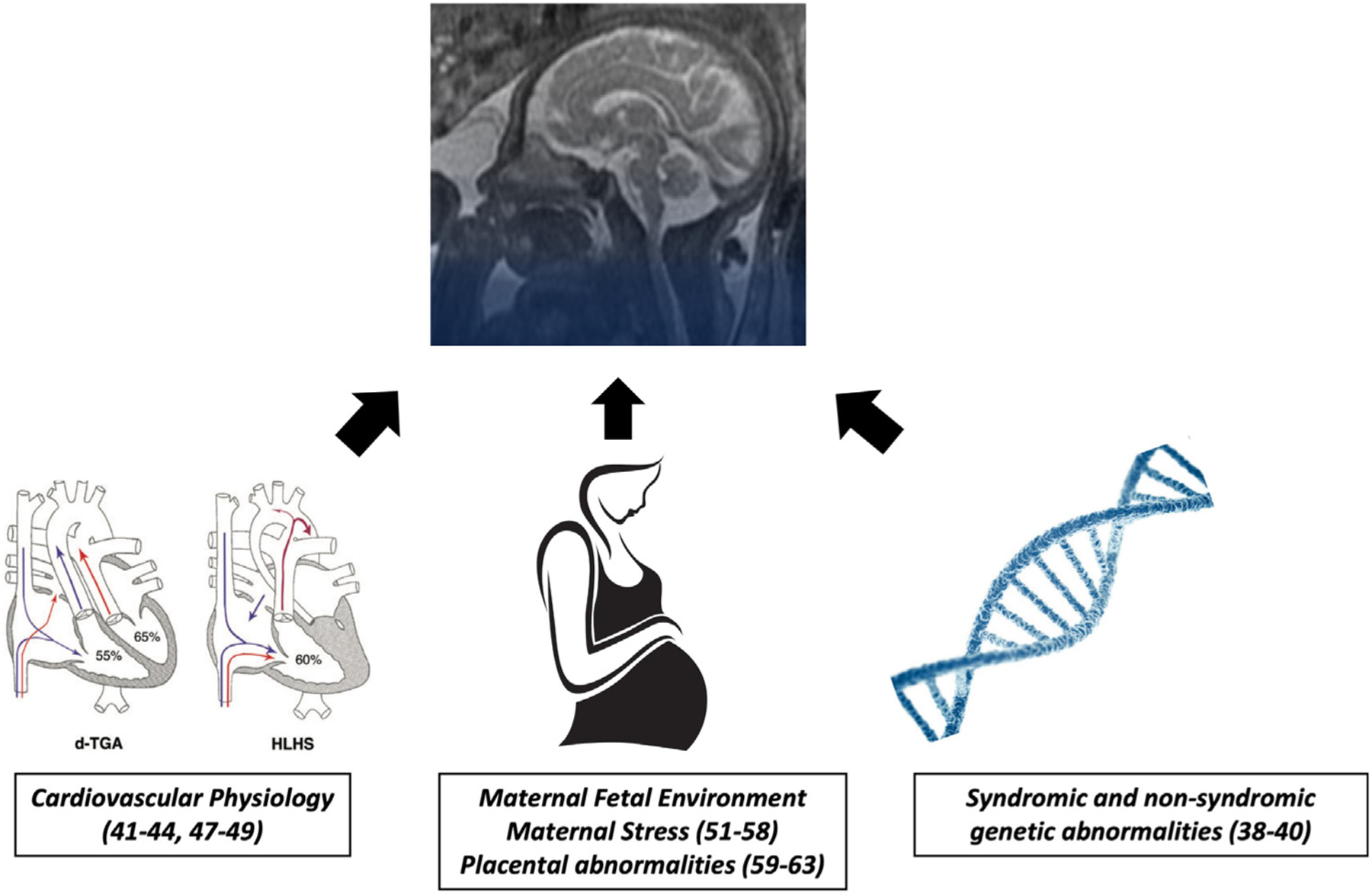
Contributors to delayed brain development *in utero*. The etiology of prenatal delayed brain development in congenital heart disease is thought to be multifactorial with contributions from cardiovascular physiology, genetic differences, and an adverse maternal-fetal environment. d-TGA, dextro-transposition of the great arteries; HLHS, hypoplastic left heart syndrome.

The fetal period represents a unique time of both vulnerability and opportunity for improving ND outcomes in CHD. Circulatory abnormalities may reduce oxygen and substrate delivery to the brain just as oxygen and nutrient demands are increasing and the foundation is laid for long-term brain structure and neurodevelopment. Direct circulatory disturbances, differences in genetics, prenatal stress, and placental function may all affect brain development (Fig. 3). Yet, as understanding increases of the mechanisms of abnormal brain development, these data reveal potential strategies to intervene and improve outcomes even before birth.

Several neuroprotective strategies are at various stages of investigation. Nonhuman animal models of fetal hypoxia suggest that tetrahydrobiopterin may protect the fetal brain by improving oligodendrocyte maturation and cerebral myelination.^61^ Ongoing trials in pregnant woman with fetuses diagnosed with CHD or in the early neonatal period are studying acute maternal hyperoxygenation (Acute Maternal Hyperoxygenation in CHD; NCT03944837), progesterone (Randomized Trial of Maternal Progesterone Therapy; NCT02133573), and allopurinol to provide protection to the vulnerable developing brain. Studies of related conditions, such as fetal distress, intrauterine growth restriction, and perinatal hypoxia-ischemia, suggest additional potential avenues, such as melatonin or caffeine.^62,63^ Finally, recent data on prenatal stress suggest that behavioural approaches to reduce stress in pregnant women with fetal CHD may provide a complementary approach to improving outcomes.^64^

In conclusion, vigourous research over the last decade has shown that multiple factors can contribute to delays in brain development beginning *in utero* for pregnancies affected by CHD. Brain immaturity in turn heightens the postnatal susceptibility to brain injuries and impaired ND outcomes coupled with other clinical factors in the postnatal time period. Thus, targeting interventional strategies to optimise brain growth and maturity *in utero* may have long-lasting beneficial effects by decreasing the susceptibility to postnatal inciting factors that further contribute to adverse ND outcomes.

## Figures and Tables

**Table 1.** Common brain abnormalities noted on MRI among fetuses and/or neonates with complex congenital heart disease

Domain: MRI modality	Abnormality
Qualitative brain development: visual inspection	Ventriculomegaly/asymmetry^22^ Dysmature myelination^13,65^ Delayed sulcation^13,65^
Brain volumes: morphometry	↓ Global and regional brain volumes^14,19,21,23^
Microstructural brain development: diffusion tensor imaging	↑ Apparent diffusion coefficient^12^ ↓ Fractional anisotropy^12^
Metabolic brain development: spectroscopy	↓ *N*-acetyl aspartate–choline ratio^14^ ↑ Lactate^14^
Oxygenation/nutrient delivery: Functional MRI, cardiovascular MRI	↓ Oxygen consumption^44^ ↓ Oxygen delivery^44^ ↓ Tissue oxygenation^27,28^

↓, Decreased; ↑, Increase. MRI, magnetic resonance imaging.
